# Is it feasible for surgical trainees to acquire JAG endoscopy accreditation by CCT? National online survey of UK trainees

**DOI:** 10.1007/s00384-025-05058-7

**Published:** 2026-01-03

**Authors:** Adil N. Ahmad, Shafquat Zaman, Adewale Ayeni, Sauid Ishaq, Peter Waterland, Prajeesh Kumar, Sarah Mills, Akinfemi Akingboye

**Affiliations:** 1 Worcestershire Acute Hospitals NHS Trust, Worcestershire Royal Hospital, Charles Hastings Way, Worcester, UK; 2 School of Medical Sciences, College of Medicine and Health, University of Birmingham , Edgbaston, Birmingham, B15 2TT UK; 3 The Dudley Group NHS Foundation Trust, Russells Hall Hospital, Dudley, West Midlands, UK; 4 Sandwell and West Birmingham Hospitals NHS Trust, West Bromwich, West Midlands, UK; 5 Chelsea and Westminster Hospital, London, UK; 6 Imperial College London, London, UK; 7 Life Sciences and College of Medicine, Aston Medical School, Aston University, Birmingham, UK

**Keywords:** Higher surgical training, Endoscopy, COVID-19 pandemic, Electronic questionnaire

## Abstract

**Background:**

Higher surgical trainees often struggle to attain endoscopy competencies. We aimed to obtain a national picture of higher surgical trainees’ endoscopy experience, highlight barriers to training, and explore potential solutions.

**Methods:**

A 40-point electronic questionnaire was designed and disseminated to higher surgical trainees across the UK. Anonymous responses were collected and recorded from 26/10/2020 to 11/06/2021.

**Results:**

A total of 139 higher surgical trainees from 16 out of the 19 regional UK deaneries responded. 75.9% (82/108) had some endoscopy training, and 19.4% (21/108) had no endoscopic training. 27.8% (30/108) had performed over 200 procedures. 77.8% (105/135) were not made aware of endoscopy training requirements by their Training Programme Directors (TPDs). 59.6% (65/109) had no named endoscopy supervisor. Only 49.1% (53/108) felt supported by their endoscopy trainers. Joint Advisory Group on GI Endoscopy (JAG) certification was infrequent, and the highest levels, 14.4% (15/104), were achieved in oesophagogastroduodenoscopy (OGD). Only 55.8% (24/43) of JAG-certified trainees felt competent in that procedure. 50.0% (7/14) of ST8 (final year trainee) respondents were not JAG certified in any procedure. 90.6% (96/106) faced challenges in gaining endoscopy training. The most common obstacles were the COVID-19 pandemic 87.9% (94/107), on-call commitments 80.2% (85/106), lack of allocated endoscopy sessions 80.2% (85/106), insufficient endoscopy training lists 76.4% (81/106), and competition with non-surgical trainees 64.2% (68/106).

**Conclusions:**

Our survey provides detailed evidence of the challenges faced by surgical trainees in gaining endoscopy training. Suggested solutions include allocated endoscopy trainers, dedicated endoscopy-only training blocks, and early guidance about endoscopy training and certification.

**Supplementary Information:**

The online version contains supplementary material available at 10.1007/s00384-025-05058-7.

## Introduction

Previous literature has highlighted the challenges of endoscopy training during the COVID-19 pandemic [1, 2]. This paper provides detailed evidence of the experience of higher surgical trainees in endoscopy from their perspective and the extent of the problem with obtaining training prior to CCT. It also suggests tangible solutions going forward.

### Higher surgical training in the UK

Higher surgical trainees in the UK are expected to gain a wide range of competencies by the time they attain their Certificate of Completion of Training (CCT), which for most trainees occurs at the end of a 6-year training programme (ST3–ST8). In the first 4 years (ST3–ST6), the focus is on general surgery. At the end of the fourth year of training (ST6), trainees are expected to declare their choice of sub-speciality. This is followed by 2 years of focused sub-speciality training (ST7–ST8). 

The Joint Committee on Surgical Training (JCST) General Surgery Curriculum of 2016 [3] set out target competencies in detail. Alongside on-call commitments, emergency/elective operative proficiency with targets in both open and laparoscopic surgeries, outpatient clinics, teaching, post-graduate examinations, courses, participating in clinical audits, research, and management roles, trainees aiming to certify in upper gastrointestinal (UGI) or colorectal surgery can also aim to achieve competencies in endoscopy.

From August 2021, the JCST General Surgery Curriculum [4] has shifted the focus towards competency-based assessment. Certification in colonoscopy is not a requirement for CCT in general surgery, although trainees aspire to obtain it as it is often a desired (if not essential) part of the person specification in applying for consultant posts.

### Governance of endoscopy training in the UK

In the UK, endoscopy training is overseen by the Joint Advisory Group on Gastrointestinal Endoscopy (JAG) [5], with surgical, medical, and allied discipline societies all being stakeholders. All endoscopy trainees are expected to meet and are assessed on, a series of evidence-based objective endpoints. The progress of an individual’s endoscopy training is tracked via the online JAG Endoscopy Training System (JETS) database. An indicative number of procedures is suggested (e.g. 200 colonoscopies), but ultimately, certification is provided after an official independent summative assessment.

### The impact of the COVID-19 pandemic on training

The recent COVID-19 pandemic has significantly impacted elective surgery [6], and thus higher surgical training [7], along with its impact on endoscopy provision. In light of the pandemic, the British Society of Gastroenterology (BSG) and JAG published a joint statement recommending ‘restricting numbers of staff in rooms for all procedures – e.g. limit trainees’ [8]. Staff redeployment and the cancellation of endoscopy courses during the pandemic had further restricted limited training opportunities.

Anecdotal evidence suggests that these factors mean that higher surgical trainees often struggle to meet their endoscopy competencies in a timely fashion. We wanted to investigate this by documenting evidence of the UK higher surgical trainee experience in endoscopy and highlight the barriers and potential solutions to this challenge.

## Methods

We designed a 40-point voluntary questionnaire on www.surveymonkey.com (Momentive Inc. San Mateo, CA, USA)—a platform which is frequently used in the healthcare setting to collect and evaluate quantitative/qualitative data. Using purposive sampling, this cross-sectional research was aimed at UK higher surgical trainees (ST3–ST8). The questionnaire was preceded by a statement explaining its purpose. The survey was de-identified so that program directors and chairs would not have access to the responses. Respondents were not incentivised for their participation in this study. IP addresses were checked and confirmed to ensure no duplicate responses.

The study was conducted according to the Checklist for Reporting Results of Internet E-Surveys (CHERRIES) [9] (Appendix A) and the STROCSS 2021 guidelines [10] (Appendix B). Furthermore, it was registered on Research Registry [11] (ID: researchregistry8102).

A survey link was disseminated via email to all higher surgical trainees in deaneries across the UK, via deanery heads or through individual lead trainees. The survey was also disseminated via the Association of Coloproctology of Great Britain and Ireland (ACPBGI) and the Association of Laparoscopic Surgeons of Great Britain and Ireland (ALSGBI).

The survey questions were developed after a comprehensive review of pertinent issues affecting endoscopy training in the UK, by both trainers and trainees before converting them to an online format and beta testing to ensure brevity, clarity, relevance, and consistent interpretation. The survey was then refined, incorporating feedback from the beta testing. The final survey design was completed on 26/10/2020.

Our questionnaire was designed using both nominal responses and Likert scales to ensure comprehensiveness and ease of analysis. Furthermore, the construct of the questionnaire was designed in a non-adaptive format to prevent ambiguity.

This study did not require prior ethical approval. It was done as a quality improvement initiative to gain insight into endoscopy training in general surgery in the UK.

Invitational emails to prospective respondents were specifically sent through the School of Surgery and relevant surgical training associations, to ensure confidentiality. Data compilation and storage was done through a dedicated NHS hospital-based computer which only the investigators had access to, with full compliance with GDPR guidance.

Anonymous responses were collected prospectively between 26/10/2020 and 11/06/2021. The planned target response number was 100. Subsequent analysis of results was performed using tools within www.surveymonkey.com.

### Inclusion criteria

- Higher general surgical trainees (ST3–ST8)
- Post-CCT trainees up to, and including, those in their first year after completion of training
- Trainees with a sub-speciality interest in upper GI, colorectal, hepatobiliary, endocrine, and breast surgery were included.

### Exclusion criteria

- Core surgical trainees (CT1–CT2)
- Non-training surgical grades

## Results

### Demographics

There were 139 respondents in total with representation from 16 of the 19 training deaneries from across the UK. The most represented deaneries were the West Midlands (26.5% = 35/132), London (18.9% = 25/132), and Yorkshire and Humber (15.9% = 21/132).

**Fig. 1:**
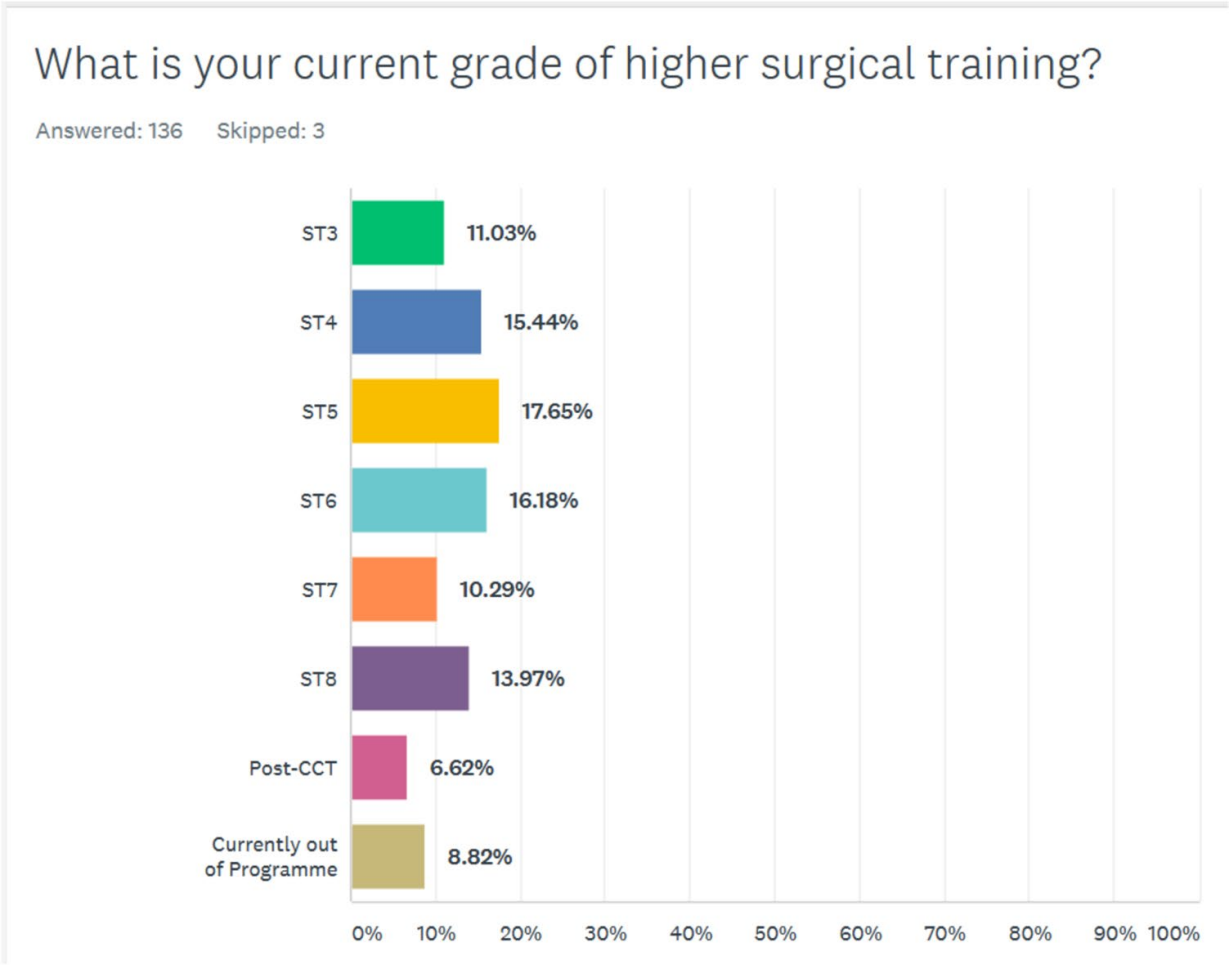
Grade of higher surgical training

There were no core surgical trainees or non-training surgical grades amongst the respondents. Approximately 74.8% (104/139) of participants answered every question category in the survey. There was a wide range of higher surgical training grades represented amongst our respondents (Fig. 1).

### Career planning

For sub-speciality interest, 67.2% (90/134) declared an interest in colorectal surgery, 20.1% (27/134) upper GI, 5.2% (7/134) hepatobiliary, and 1.5% (2/134) breast/endocrine, and 6.0% (8/134) were undecided. 32.1% (43/134) had decided on their sub-speciality before entering higher surgical training. By the end of their first year as a higher surgical trainee (ST3), 57.5% (77/134) had made this decision. 75.2% (100/133) discussed sub-speciality choice with their assigned educational supervisor (AES).

17.2% (23/134) of respondents were not familiar with the training and certification pathway for UK endoscopists. Of those that were, 41.4% (46/111) only became aware after the completion of their first year of training (ST3).

Only 32.6% (44/135) were informed by their Training Programme Director (TPD) of the need for endoscopic training and certification at the start of training.

### Endoscopy training

**Fig. 2:**
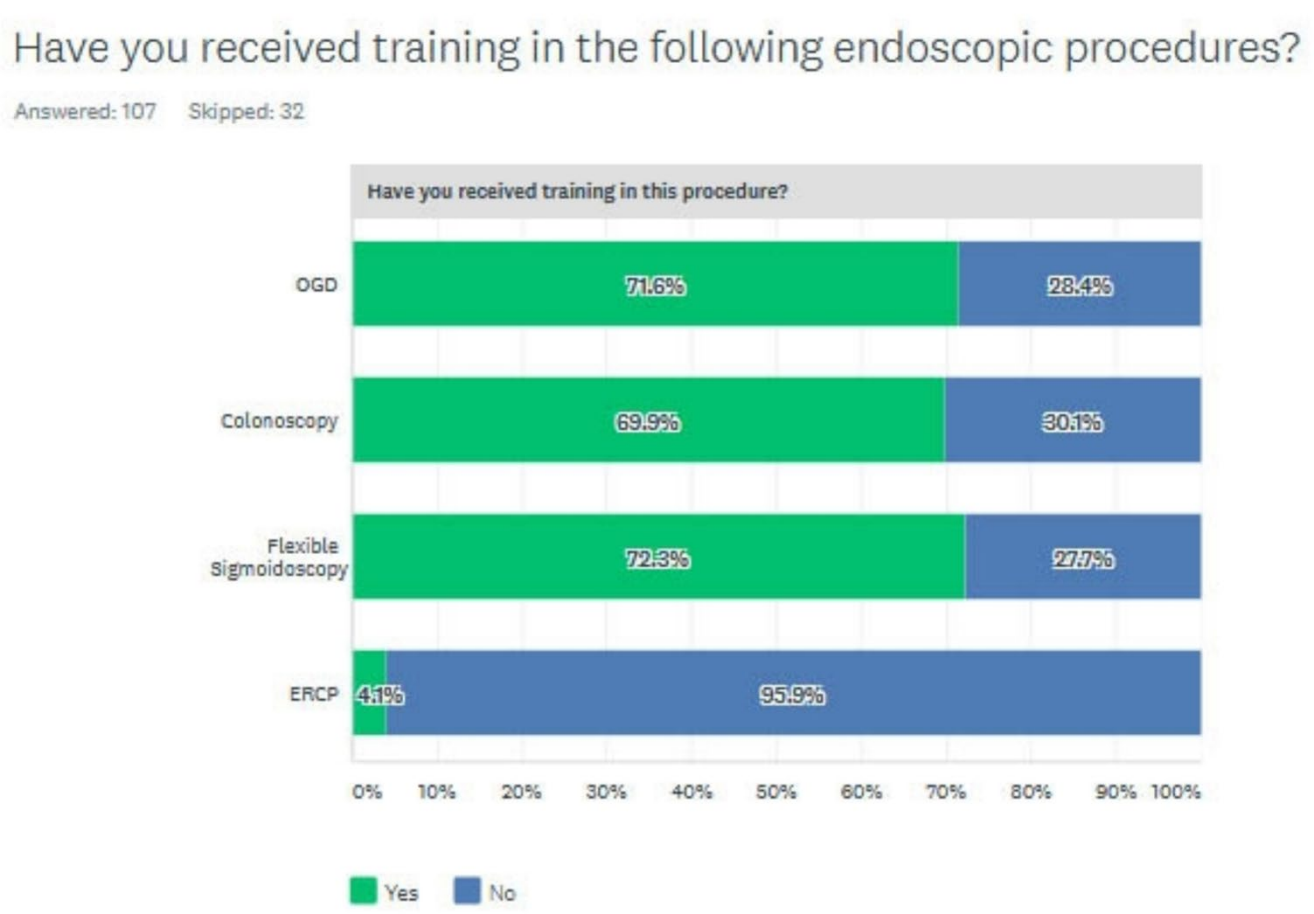
Percentage of trainees who have received training in different endoscopy modalities

Of the respondents, 75.9% (82/108) had some endoscopy training during their surgical training. 88.9% would still want endoscopy training even if it was not mandatory/part of the curriculum. 71.6% (73/102) had received training in OGDs, 69.9% (72/103) in colonoscopy, 72.3% (73/101) in flexible sigmoidoscopy, and only 4.1% (4/98) in ERCP (Fig. 2).

Diagnostic training was far more common than therapeutic: OGD (77.4% vs. 15.4%), colonoscopy (76.7% vs. 43.2%), flexible sigmoidoscopy (79.5% vs. 43.5%), and ERCP (5.0% vs. 5.3%).

**Fig. 3:**
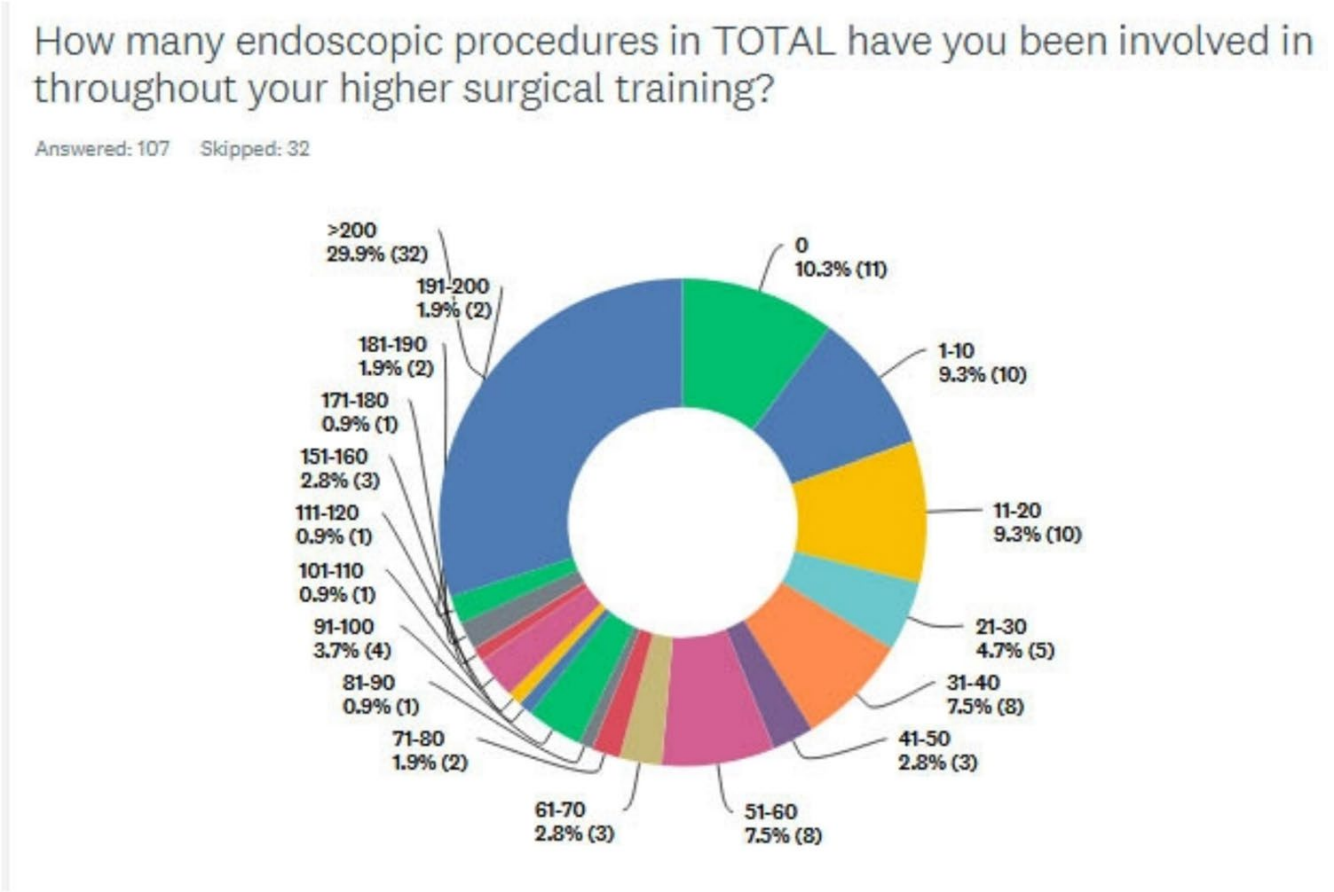
Total number of procedures recorded throughout higher surgical training

10.3% (11/107) of respondents had no experience of endoscopic procedures. 29.9% (32/107) had been involved in over 200 procedures. As shown in Fig. 3, there was a wide range of experience of respondents between these categories.

### JAG courses, training, and certification

We wanted to better understand the stage of training; trainees were completing JAG-approved endoscopy courses. 42.0% (42/100) had attended an OGD course, 48.0% (48/100) colonoscopy course, 14.3% (13/91) flexible sigmoidoscopy course, and only 1.1% (1/88) an ERCP course.

Of these, most had done so within the early part of their higher surgical training pathway (ST3–ST5). The breakdown was as follows: 85.4% (35/41) in OGDs, 82.2% (37/45) in colonoscopy, 100% (13/13) in flexible sigmoidoscopy, and 100% (1/1) in ERCP.

JAG certification in every procedure was infrequent: 14.4% (15/104) in OGDs, 7.8% (8/102) in colonoscopy, 4.3% (4/93) in flexible sigmoidoscopy, and 0% (0/87) in ERCP.

Of those not JAG-certified, we asked at what point in their training they aspired to achieve certification. Although the response rate was low, a high proportion of trainees hoped to achieve this as late as ST8 or even post-CCT: 75.0% (6/8) in OGDs, 62.5% (5/8) in colonoscopy, 71.4% (5/7) in flexible sigmoidoscopy, and 83.3% (5/6) in ERCP.

We analysed ST8 responses (final year trainees) in more detail: 50.0% (7/14) were not JAG-certified in any procedure. 8.3% (1/12) had certified in OGD, 38.5% (5/13) in colonoscopy, 30.0% (3/10) in flexible sigmoidoscopy, and 0.0% (0/10) in ERCP.

Of those managing to attain JAG certification, only 55.8% (24/43) ‘agreed’ or ‘strongly agreed’ with the statement: ‘I am competent in the endoscopic procedure for which I was certified’.

30.2% (13/43) of respondents either ‘disagreed’ or ‘strongly disagreed’ with the above statement, suggesting a need for continued mentorship in endoscopy post-certification.

### Experience of hospital endoscopy training

We looked at the quantity and quality of endoscopy training provided in UK hospitals. 59.3% (64/108) of respondents did not have a dedicated endoscopy training session scheduled in their rota. Of those that did, 47.7% (21/44) had that session less than once a week. 68.6% (72/105) had to attend non-training lists to improve or enhance their training. Of these, 32.4% had to attend these lists at least once per week, and 52.4% had to attend these lists at least once every 2 weeks.

Only 25.2% (27/107) have used simulators or alternative non-patient training methods, but of these, 85.2% did so less than once a month.

86.2% (94/109) of respondents stated that their hospital endoscopy services were JAG-accredited. 68.8% (75/109) had a JAG-approved trainer (defined as a trainer completing a JAG-approved Training the Colonoscopy Trainers Course or Training the Gastroscopy Trainers Course), whilst 23.9% (26/109) were unsure about their trainer’s status.

59.6% (65/109) did not have a named endoscopy training supervisor, and the same number did not have an induction with an endoscopy trainer prior to starting their hospital rotation.

Perhaps unsurprisingly, 59.6% (65/109) felt that surgical consultants were the most instrumental in their endoscopy training. 12.8% (14/109) thought gastroenterology (medical) consultants and 12.8% (14/109) thought nurse endoscopists were the most instrumental. Importantly, 70.4% (76/108) were not given an opportunity to train with a medical gastroenterologist.

Only 41.8% (44/108) of respondents ‘agreed’ or ‘strongly agreed’ that ‘certified endoscopic trainers were readily available’ during their training. Only half (49.1%, 53/108) ‘agreed’ or ‘strongly agreed’ that they felt supported by their endoscopy trainers.

### Challenges in endoscopy training

90.6% (96/106) of respondents faced challenges in gaining adequate endoscopy training. 87.9% (94/107) felt that the COVID-19 pandemic contributed towards this, whilst 80.2% (85/106) felt that surgical on-call commitments created difficulties in accessing regular endoscopy training sessions.

Other common challenges shared by trainees included (respondents could tick multiple boxes):

1. Lack of allocated endoscopy sessions built into their rota—80.2% (85/106).
2. Lack of endoscopy training lists—76.4% (81/106).
3. Competition for training opportunities with non-surgical trainees—64.2% (68/106).
4. Insufficient information from the training deanery on when to commence endoscopy training—49.1% (52/106).
5. Insufficient information from the deanery on the need for endoscopy training—46.2% (49/106).
6. Un-cooperative endoscopy department lead—37.7% (40/106).
7. Competition for training opportunities with trust grade colleagues—34.9% (37/106).
8. Insufficient hands-on exposure during endoscopic training—32.1% (34/106).
9. Lack of JAG-accredited endoscopy services in hospital—13.2% (14/106).

Only 25.5% (27/106) of respondents felt the number of points per endoscopy list was appropriate. 28.3% (30/106) were unsure about what points per endoscopy list referred to.

76.4% (81/106) felt that endoscopy training is not useful for surgical trainees with no interest in upper GI or colorectal surgery. The majority of respondents (76.6%, 82/107) felt that endoscopy training and certification should be mandatory as part of the higher surgical training curriculum.

**Fig. 4:**
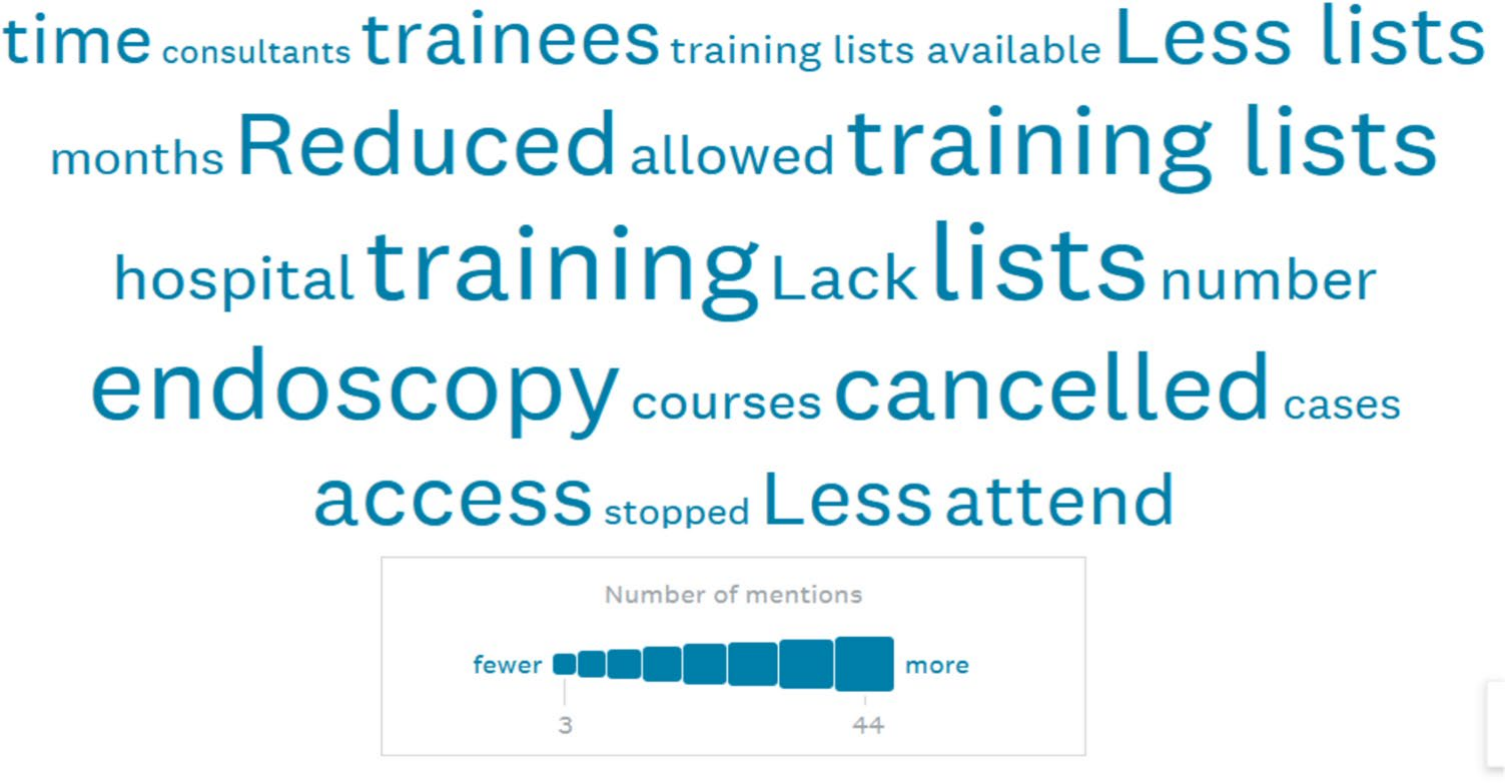
Word cloud of responses to how the COVID-19 pandemic affected endoscopy training

Finally, we asked an open question to trainees about the COVID-19 pandemic and its impact on their endoscopy training. Figure 4 shows a word cloud (tag cloud) of the 90 responses (via www.surveymonkey.com analytics). The size of the word in the picture correlates with the frequency with which it was mentioned. The common themes in the answers related to reduced or cancelled training lists, fewer trainees being allowed into lists, patients being less likely to attend appointments, and cancelled endoscopy courses.

## Discussion

### The future of endoscopy training

The challenge of providing the public with high quality, timely, and accessible diagnostic services has been brought to the fore by Professor Mike Richard’s review of diagnostic services nationally for NHS England ‘Diagnostics: Recovery and Renewal’ published in November 2020 [12]. Workforce issues are key to the delivery of these services.

As highlighted by the Royal College of Surgeons, England, ‘one thing is for sure in the uncertain COVID world: No Training Today, No Surgeons Tomorrow’ [13]. The same can be said of surgical endoscopists.

The JAG 2019 breakdown of endoscopy workforce by role showed that 27% of all endoscopy procedures performed in the UK are by surgeons, compared to 38% by medical gastroenterologists. This is a substantial contribution and must be evaluated in the context of increasing demand for endoscopy services and a dwindling endoscopy workforce, especially in bowel cancer screening [14].

Despite surgical endoscopists contributing a significant proportion of endoscopy services delivered nationally, our survey clearly demonstrates that surgical trainees face significant challenges in reaching their endoscopy competencies. With 16 of the 19 UK deaneries represented, there was excellent geographical coverage of respondents.

### Career—early planning and guidance

From our cohort, it appears that surgical trainees are making decisions about sub-speciality at an early stage of their careers. By the end of the first year (ST3), most had made up their choice of sub-specialisation; yet, the vast majority had not been informed by their TPD about the need for endoscopic training for certification. The majority were unaware of the certification pathway for endoscopists in the UK. Nearly half of trainees quoted lack of information about the need for endoscopy training or when to commence it, as an obstacle to gaining training. We acknowledge that some of the onus must be placed on the trainee themselves. However, a simple way of overcoming this would be for the deanery to provide clear advice and information on endoscopy training during the ST3 deanery induction.

### Endoscopy training—where we stand

Approximately one-quarter of our respondents have had no endoscopy training, and this is a cause for concern. Training in OGDs, colonoscopy, and flexible sigmoidoscopy was around the 75% mark, whilst ERCP or therapeutic procedures was far lower. Whilst this is expected, it is worth noting that new modalities for assessing the risk of bowel cancer and significant polyps, such as FIT, in both symptomatic and asymptomatic populations will inevitably yield more colonic pathology requiring intervention within endoscopy lists in the near future.

### JAG courses and training—are we leaving it too late?

JAG endoscopy course attendance was lower than expected, even in OGDs and colonoscopy where it was less than 50%. Cancellation of courses during the COVID-19 pandemic has contributed to this.

JAG certification in endoscopy procedures was quite low—less than 15% in OGDs and far less in other modalities. Of those who had not attained JAG certification and aspired to do so, the vast majority were hoping to achieve this by ST8 or even post-CCT. Most alarmingly, half of our ST8 respondents had not received JAG certification in any procedure. This is consistent with previous studies in the literature [15].

We advocate that trainees should ideally be certifying in these procedures much earlier and not as late as ST8, when they should be focussing on, arguably, more time sensitive goals such as exams, fellowship applications, or transitioning into the role of a consultant. Aspiring to certify post-CCT may indicate that trainees lack confidence in the higher surgical training programme to provide them with sufficient time and support to attain endoscopy competencies [16].

It is equally concerning that of those who had attained JAG certification, just over half felt competent in performing that procedure, and a third did not feel competent at all. This suggests that there may be room for further training, in the form of surgical endoscopy fellowships, or mentorship programmes post-CCT.

### Challenges in endoscopy training—solutions to attain quantity and quality

Our study has highlighted a wide range of obstacles and challenges faced by surgical trainees in gaining high quality endoscopy experience. Trainees must balance the demands of service provision with elective and endoscopy training. However, many trainees have infrequent endoscopy lists or are attending non-training lists. Previous studies have shown that sustained exposure to training is key to enhancing and improving endoscopy skills [17], and dedicated blocks of endoscopy training with no on-call or surgical commitments may be an innovative way forward. It is also worrying that most trainees neither felt supported by their trainers nor that those trainers were readily available.

### Should endoscopy training be accessible only after declaring subspeciality?

71.6% (73/102) of our respondents had received some OGD training, and yet, only 20.1% (27/134) wanted to pursue upper GI as a future subspeciality. To ration endoscopy services, one option could be to only provide endoscopy training to those that have declared subspeciality interest. However, we would strongly advise against this.

Trainees are required to declare sub-speciality interest by the end of ST6. This would leave too little time to start and complete endoscopy competencies alongside post-graduate examinations and surgical competencies in the busiest 2 years of their training. Even at present, 50% of our final year respondents had not certified in any endoscopy modality.

As endoscopy is expected to form a significant part of a Consultant Surgeon’s future NHS practice, early exposure to endoscopy training may influence a more informed future sub-speciality choice. This is reflected in the desire to gain endoscopy training by 88.9% of respondents, even if it was not mandatory or part of the curriculum. Increasing access to training from nurse endoscopists, most of whom become proficient at OGD prior to colonoscopy, is part of the reason why more trainees have had OGD training than other modalities.

## Recommendations

### Appropriate endoscopy lists

We strongly advocate for all trainees to have at least one regular (half-day) training list with a trainer who has attended the relevant JAG Training the Trainers Course. Overbooked lists add pressure upon the trainer and staff, which adversely impacts the training experience and quality. It is important that training lists have appropriate levels of points assigned to them to facilitate training. Whilst simulation has been shown to be a useful adjunct [18–20], it should not be a replacement for high-quality, patient-focused endoscopy training.

### Supervision and endoscopy training programme directors

Trainees should have a named endoscopy trainer within their hospital overseeing their progress and providing regular feedback and assessment. This should be mandated as a part of the Personal Development Plan and initial Assigned Educational Supervisor meeting to create accountability on the part of the trainee, trainer, and the hospital trust.

There should be endoscopy TPDs from the deanery to oversee endoscopy training allocations for the surgical trainees. Where trainees fall behind, the endoscopy TPD should address this with both the trainee and their institution as part of their Annual Review of Competence Progression (ARCP).

### Endoscopy training blocks

We would urge regional TPDs to embrace the concept of endoscopy training blocks, where trainees are able to immerse themselves in training for a few weeks at a time without the pressure of on-calls, clinics, or elective surgical lists. This is likely to lead to more rapid acquisition of endoscopic skills, a significantly smaller chance of such training being cancelled, and higher satisfaction amongst trainees.

### Is There a challenge of interdisciplinary endoscopy training?

Unfortunately, in this study, 70.4% of surgical trainees struggled to gain access to training from medical gastroenterologists. With surgical and medical societies being equal stakeholders in JAG, this should ideally do away with such inter-disciplinary obstacles, but this ideal is far from the reality demonstrated. Clearly, there are wonderful examples in units across the country where this is not an issue. However, whilst it is important not to create a surgical versus medical dichotomy, in order to address this problem where it exists in the short term, we must ensure that there are sufficient surgical trainers who have attended relevant courses to meet the requirements of the number of trainees at a particular unit. This includes not only surgical trainees but non-surgical trainees and trust-grade colleagues, whose access to endoscopy training must be facilitated in a thoughtful way that does not impact trainees’ attainment of competencies in the short time they have available.

As the American Society of Gastrointestinal Endoscopy (ASGE) has highlighted, ‘teaching faculty should not only be expert endoscopists who are committed to the entire training process…[but] training directors need to ensure that an adequate number of such individuals are available to ensure quality teaching…to ensure that the standards are maintained’ [21].

### Re-framing endoscopy training for surgical trainees

Making these changes requires a cultural shift in the way that endoscopy training is viewed for surgical trainees. Workforce pressures mandate that endoscopy training for surgical trainees cannot be an afterthought. Endoscopists may be in short supply in the future [22], and surgeons must continue to be important stakeholders. We recognise that the new surgical curriculum of 2021 is a good start along with JAG guidance [23].

The national rollout of Endoscopy Academies and the interim role of Endoscopy Immersion Fellowships will hopefully play a useful role in further improving access to endoscopy training for surgeons. These initiatives will allow them to continue to play a vital role in the endoscopy workforce, delivering high-quality care to increasingly complex patients [24].

## Summary of recommendations

A structured modular approach to endoscopy training should be implemented deanery-wide, incorporating expertise from advanced nurse endoscopists, gastroenterologists, and consultant surgeons. The use of immersive training blocks will enhance a trainees’ proficiency in performing endoscopic procedures and work towards meeting criteria for certification. Intensive therapeutic endoscopy sessions should help to refine advanced skills for higher surgical trainees.

Joint endoscopy leads from surgery and gastroenterology should oversee list scheduling to ensure equitable access to training. Additionally, the local training deanery should fund basic JAG courses, reducing financial barriers.

Moreover, regular protected training lists are encouraged in addition to utilising ad hoc training opportunities.

## Broader implications of study findings

Our findings highlight the critical challenges present in current surgical endoscopy training with broader implications for policymakers, educators, and stakeholders. Early (ST3 level) and clear guidance on endoscopy certification requirements is essential to help trainees formulate appropriate and timely career plans and prevent last-minute efforts to achieve competencies, which can compromise quality. Policymakers should mandate structured endoscopy training, including named supervisors and regular, protected training lists led by certified trainers. Implementing dedicated endoscopy training blocks free from on-call duties and surgical commitments would allow trainees to gain sustained, high-quality experience, improving competence and confidence.

Educators must foster interdisciplinary collaboration, ensuring surgical trainees can access training from gastroenterologists and advanced nurse endoscopists. Additionally, increasing the use of simulation and alternative training methods can supplement patient-based learning and improve technical skills in a controlled environment. Stakeholders should also invest in trainer development, ensuring an adequate number of certified trainers are available to support increasing trainee numbers and demand.

Given that surgical endoscopists contribute to national endoscopy services, failure to address these barriers risks exacerbating workforce shortages, especially in cancer diagnostics. A cultural shift is needed to prioritise endoscopy training within surgical programmes, recognising its crucial role in future consultant practice. Timely interventions now will secure a skilled, sustainable workforce capable of meeting growing service demands.

## Limitations

The aim of this study was to provide comprehensive and detailed evidence rather than to scratch the surface of what is clearly a complex challenge. Therefore, our survey had a very large number of questions (40). To mitigate for a potentially large attrition rate of respondents giving up on the survey, we made all questions non-mandatory. Hence, the raw number of responses sometimes varied per question, but not significantly. Absolute numbers and percentages are provided for clarity.

We acknowledge that our survey sample may not represent all higher surgical trainees in the UK as the questionnaire was likely not disseminated to all eligible trainees and participation was voluntary. Using different platforms for survey dissemination means it is difficult to give an accurate response rate. However, we believe that our results accurately represent the views of those who chose to participate and that our interpretations are likely reflective of wider patterns of UK surgical trainee experience.

There were a higher proportion of respondents interested in colorectal surgery, in part because of dissemination through the ACPBGI. Nevertheless, we did have respondents interested in upper GI surgery, hepatobiliary, endocrine, and breast surgery.

We recognise that this survey was conducted during the COVID-19 pandemic when endoscopy training was adversely impacted and hence trainee experience may appear worse. However, aside from the COVID-19 pandemic, this survey revealed a wide range of factors that surgical trainees identified as obstacles to gaining their endoscopy competencies.

## Conclusion

Our survey provides detailed evidence of the challenges faced by surgical trainees in gaining endoscopy training and meeting criteria for JAG certification. Suggested solutions include allocated endoscopy trainers, dedicated endoscopy-only training blocks, and early guidance about endoscopy training and certification.

## Supplementary Information

Below is the link to the electronic supplementary material.

ESM 1 (DOCX 20.7 KB)

ESM 2 (DOCX 34.5 KB)

## Data Availability

The data presented in this study are available on request from the corresponding author.

## Declarations

### Permission to reproduce material from other sources

No other sources were used in this study. References have been listed.
