# Supplementary material for: Is it feasible for surgical trainees to acquire JAG endoscopy accreditation by CCT? National online survey of UK trainees
Source: Int J Colorectal Dis. 2026 Jan 3;41(1):13. doi: 10.1007/s00384-025-05058-7 (PMC12764598; doi:10.1007/s00384-025-05058-7)
Supplement: Supplementary file 1 — (DOCX 20.7 KB) [file 384_2025_5058_MOESM1_ESM.docx]

**Appendix A - Checklist for Reporting Results of Internet E-Surveys (CHERRIES)**

| ***Checklist Item*** | ***Explanation*** | ***Page Number*** |
| --- | --- | --- |
| Describe survey design | The target population was UK higher surgical trainees (ST3-ST8). | 6 |
| IRB approval | This study did not require prior ethical approval. It was done as a quality improvement initiative to gain insight into endoscopy training in general surgery in the UK and did not involve patients or the public. | 7 |
| Informed consent | Invitational emails to prospective respondents were specifically sent through the School of Surgery and relevant surgical training associations. In these emails, it was clearly specified what the aim of the study was and who the lead investigator was. | 7 |
| Data protection | The survey was de-identified so that program directors and chairs would not have access to the responses. The process of data compilation and storage was done through a dedicated NHS hospital-based computer which only the investigators had access to and was in full compliance with GDPR guidance. | 7 |
| Development and testing | We designed a 40-point voluntary questionnaire on [www.surveymonkey.com](http://www.surveymonkey.com) (Momentive Inc. San Mateo, California, USA) – a platform which is frequently used in the healthcare setting to collect and evaluate quantitative/qualitative data. The survey questions were developed after a comprehensive review of pertinent issues affecting endoscopy training in the UK. The appropriateness of the questions generated were reviewed by both trainers and trainees before converting them to an online format and beta testing to ensure brevity, clarity, relevance, and consistent interpretation. The survey was then refined, incorporating feedback from the beta testing. | 5-6 |
| Open survey versus closed survey | This was technically an open survey; however the links were sent out to the target population via email invitations. | 6-7 |
| Contact mode | A survey link was disseminated via email to all higher surgical trainees in deaneries across the UK, via deanery heads or through individual lead trainees. The survey was also disseminated via the Association of Coloproctology of Great Britain and Ireland (ACPGBI) and the Association of Laparoscopic Surgeons of Great Britain and Ireland (ALSGBI). | 6-7 |
| Advertising the survey | As above – it was sent via email. | 6-7 |
| Web/E-mail | Responses were all collected via [www.surveymonkey.com](http://www.surveymonkey.com). | 5, 7 |
| Context | Not published on a website. Only sent out via email. | 6-7 |
| Mandatory/voluntary | It was a voluntary survey. We decided not to make any of the questions mandatory. | 5, 19 |
| Incentives | Respondents were not incentivised for their participation in this study. | 6 |
| Time/Date | Anonymous responses were collected prospectively between 26/10/2020 to 11/06/2021. | 7 |
| Randomization of items or questionnaires | Randomisation was not required or applicable to our study. Responses were anonymous and de-identified. | 6-7 |
| Adaptive questioning | The construct of the questionnaire was designed in a non-adaptive format to prevent ambiguity. | 6 |
| Number of Items | There were 40 questions all displayed on one page that required respondents to scroll down. | 5 |
| Number of screens (pages) | There were 40 questions all displayed on one page that required respondents to scroll down. | 5 |
| Completeness check | We decided not to make any of the questions mandatory. | 19 |
| Review step | Respondents were able to change their answers until pressing on the submit button. | 5 |
| Unique site visitor | All respondent IP addresses were checked and confirmed to ensure there were no duplicate responses | 6 |
| View rate (Ratio of unique survey visitors/unique site visitors) | We did not calculate view rates as this was done via an invitational link directly to the survey. | 19 |
| Participation rate (Ratio of unique visitors who agreed to participate/unique first survey page visitors) | We did not calculate the participation rate as this was done via an invitational link directly to the survey | 19 |
| Completion rate (Ratio of users who finished the survey/users who agreed to participate) | 100% of those who filled in the first question, filled in the last question. However, we did not make all questions mandatory so 74.8% (104/139) of participants answered every question category in the survey. | 8 |
| Cookies used | Cookies were not used, but IP address checks were used. | 6 |
| IP check | All IP addresses were checked to prevent duplication and no two respondents used the same IP address. | 6 |
| Log file analysis | N/A | N/A |
| Registration | Our research was retrospectively registered on the Research Registry on <https://www.researchregistry.com/browse-the-registry#home/> with the Unique Identifying Number (UIN) of researchregistry8102 | 6 |
| Handling of incomplete questionnaires | All data entered was analysed. None of the questions were mandatory. | 7 |
| Questionnaires submitted with an atypical timestamp | Once the first email was sent out, all responses were included and analysed. | 7 |
| Statistical correction | No statistical correction was used or deemed necessary. | N/A |
